# Energy expended during horizontal jumping: investigating the effects of surface compliance

**DOI:** 10.1242/bio.20148672

**Published:** 2014-08-22

**Authors:** Samuel R. L. Coward, Lewis G. Halsey

**Affiliations:** 1 School of Biosciences, University of Birmingham, Edgbaston, Birmingham B15 2TT, UK; 2 Centre for Research in Ecology, Department of Life Sciences, University of Roehampton, London SW15 4JD, UK

**Keywords:** Horizontal jumping, Metabolic rate, Jumping kinematics, Surface compliance

## Abstract

We present the first data on the metabolic costs of horizontal jumping in humans, using this tractable model to explore variations in energy expenditure with substrate properties, and consider these findings in light of kinematic data. Twenty-four participants jumped consistently at the rate of 1 jump per 5 s between opposing springboards separated by either a short (1.2 m) or long (1.8 m) gap. Springboards were either ‘firm’ or ‘compliant’. Respiratory gas exchange was measured using a back-mounted portable respiratory gas analyser to represent rate of energy expenditure, which was converted to energy expenditure per metre jumped. Video data were recorded to interpret kinematic information. Horizontal jumping was found to be between around 10 and 20 times the energy cost of cursorial locomotion per unit distance moved. There is considerable evidence from the data that jumping 1.8 m from a compliant springboard (134.9 mL O_2_ m^−1^) is less costly energetically than jumping that distance from a firm springboard (141.6 mL O_2_ m^−1^), albeit the effect size is quite small within the range of compliances tested in this study. However, there was no evidence of an effect of springboard type for jumps of 1.2 m. The kinematic analyses indicate possible explanations for these findings. Firstly, the calf muscle is likely used more, and the thigh muscles less, to take-off from a firm springboard during 1.8 m jumps, which may result in the power required to take-off being produced less efficiently. Secondly, the angle of take-off from the compliant surface during 1.8 m jumps is closer to the optimal for energetic efficiency (45°), possible due to the impulse provided by the surface as it returns stored energy during the final stages of the take-off. The theoretical effect on energy costs due to a different take-off angle for jumps of only 1.2 m is close to negligible.

## INTRODUCTION

Research into human jumping has in the main determined optimum technique, maximising performance in sporting activities. For example, studies have described the conditions for achieving maximum jump distance in the standing (Wakai and Linthorne, 2005) and running long jump (Alexander, 1991; Linthorne et al., 2005), based on both empirical and theoretical calculations. Calculations of energy use during jumping take-off have been made via inverse dynamics, for example the work done by individual muscles and the work done about the joints within the lower limb have been calculated during human one-legged jumping (Jacobs et al., 1996), and hopping on a variety of surfaces (Farley et al., 1998; Ferris and Farley, 1997; Moritz and Farley, 2005), and also in jumping goats (Carroll et al., 2008). The mechanical power of vertical jumping has been calculated from first principles and models in humans (Bosco et al., 1983; Nagano et al., 2005), and the costs of leaping similarly calculated for saltatory primates (Warren and Crompton, 1998). Additionally, estimates have been made of the energy from impulse required during take-off in a number of animals including locusts (Bennet-Clark, 1975) and fleas (Bennet-Clark and Lucey, 1967). However, to the best of our knowledge, to date the cost of jumping in humans has not been measured directly. Furthermore, such data could be particularly relevant to arboreal locomotion in tree-dwelling primates (e.g. Thorpe et al., 2007).

In the present study we measured the energy cost of horizontal jumping in participants to and from both firm and compliant springboards, and for relatively short (1.2 m) and relatively long (1.8 m) jumps. Indirect calorimetry was used to measure these costs while participants repeatedly undertook horizontal jumps, at a rate of one jump every five seconds, until physiological steady state was achieved. Verification that primarily aerobic metabolic pathways were utilised was provided primarily from measurements of respiratory exchange quotients. The measurements obtained therefore provided fairly accurate estimates of the energy costs to jump horizontally and in particular are suitable for comparing the relative costs of jumping under different conditions.

We hypothesised that jumping from a compliant urface would be less costly than jumping from a firm surface because compliant surfaces can act as external energy stores that rapidly return stored energy just prior to take-off, somewhat akin to a catapult. This concept is to some extent analogous to the way that tendons store energy during walking (Cavagna and Legramandi, 2009); energy is slowly stored in these elastic tissues just prior to that energy being rapidly returned (Ishikawa et al., 2005). Key muscles contract isometrically during walking (Fukunaga et al., 2001) and thus operate around the highest force region of the force–velocity curve, permitting the most efficient use of the muscle to support body weight (Hill, 1938) during the push-off phase and thus reduce energy cost. We also hypothesised that the difference in energetic cost between jumping from a firm and from a compliant surface would be more pronounced for the longer jumps as the larger impulse required for longer jumps would allow the participants more scope to utilise the elastic energy storage of the compliant springboard. Finally we hypothesised that landing on a compliant surface would be more energetically efficient than landing on a firm surface as the compliant surface would store energy before returning it and the inherent inefficiencies in energy transfer would dissipate some of the energy prior to returning it to the participant. Conversely landing on a firm surface would require all the energy to be absorbed by the musculoskeletal system via active elongation of the muscle tendon units.

## MATERIALS AND METHODS

The experiments undertaken in this study received prior approval from the Ethics Committees of the Universities of Roehampton and Birmingham. After completing a standard screening questionnaire and a consent form to rule out participants with musculoskeletal and neurological disorders, twenty four participants (mean mass ± SD: 74.9±8.9 kg) undertook a battery of seven horizontal jump tests (conditions). These conditions represented combinations of two types of surface and two jumping distances. Jumping from a ‘compliant’ surface to a compliant surface, from a ‘firm’ surface to a firm surface, compliant to firm and firm to compliant were all undertaken for jumps of 1.8 m (the distance between the near edges of the springboards). Jumping from a compliant surface to a compliant surface, firm to firm and compliant to firm were all also undertaken for jumps of 1.2 m. Data collection was undertaken in the Slater gymnasium at the University of Birmingham, UK. During the tests eight of the participants were filmed with a mounted video camera (DCR-SR90, Sony, Japan).

Participants jumped between springboards for at least three minutes, at a frequency of one jump every 5 s guided by an electronic metronome. Rate of oxygen consumption (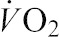) was measured via a mobile respiratory gas analyser (Oxycon mobile, Viasys, Germany). At the end of each period of activity, participants were asked to state their relative perceived exertion (RPE) (Borg, 1970; Chen et al., 2002) towards the end of the activity. The participants were mainly metabolising aerobically, demonstrated by respiratory exchange ratios typically below 1, and RPE scores typically below 16 (Scherr et al., 2013). Either the last 30 s or, more typically, 60 s of data in each condition were used to calculate mean 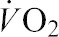 during the activity, since during this period 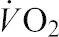 was confirmed to have plateaued indicating that the body had reached a respiratory steady state, normally after around 2 minutes as is typical for fit individuals (Chilibeck et al., 1996; Whipp and Wasserman, 1972). 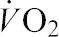 was converted to rate of oxygen consumption per metre (mL O_2_ m^−1^) to represent energy expenditure.

Springboards were considered to provide either a ‘compliant’ surface (stiffness  =  22.7 N mm^−1^, damping ratio  =  0.07) or were made ‘firm’ by inserting a wooden chock between the top and bottom surfaces (stiffness  =  881.3 N mm^−1^, damping ratio  =  0.13). The stiffness was assessed by applying a load via a known mass (92.6 kg) and measuring the displacement of the surface. Damping ratio and stiffness calculations are presented in supplementary material Fig. S1.

Video data for some participants and conditions were recorded from a camera set orthogonally to the direction of the participants' horizontal movement. Prior to a battery of tests a calibration cube of known dimensions was placed within the test area allowing a scale and aspect ratio to be applied to the video data. The video files were converted into an image stack using Virtual Dub (Version 1.6.19, http://www.virtualdub.org) and subsequently digitised using Didge (Version 2.30b1, http://biology.creighton.edu/faculty/cullum/Didge).

The videos for the jumping trials were analysed during five jumps within the last minute of each trial. The following locations were digitised: the top surface of the springboard at its free end and the participant's sacrum (used to approximate the centre of mass; COM), the head of the first metatarsal bone (toe), the centre of the lateral malleolus (ankle), lateral epicondyle of the femur (knee), greater trochanter (hip), head of the humerus (shoulder) and the styloid process of the ulna (wrist).

### Data analysis

From these digitised points the following kinematic measures were calculated: (i) duration of force application during the jump, defined as the time between the first visible application of loading to the springboard and the time at which the participant loses contact with the board; (ii) peak deflection of the springboard defined as the difference between the height of the springboard's top surface under the participant's weight prior to the jump and its minimum height during the force application; (iii) the hip, knee and ankle angles at the onset of the jump, at the point when the COM reached its lowest position during the force application (peak flexed position) and at the loss of contact with the board (start of the flight phase); (iv) peak height gained by the COM as the difference in the COM height between its peak height during the flight phase and its standing height prior to the jump; (v) the swept angles made by the arms during the jump, defined as the angles between a line from the shoulder to the wrist from: the start position to the maximum rotation of the arms in the posterior direction and the maximum posterior position to the maximum anterior position; (vi) an estimate of the take-off speed of the participant expressed as the resultant of the vertical velocity, calculated via Eqn 1, and the horizontal velocity, calculated as the horizontal distance moved by the COM during the flight period divided by the duration of the flight period.
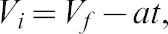
(1)where *V_i_*  =  initial vertical velocity (m s^−1^), *V_f_*  =  final vertical velocity (0 m s^−1^ at peak height), *a*  =  acceleration due to gravity (m s^−2^), and *t*  =  time from take-off to peak height (s).

**Fig. 1.**
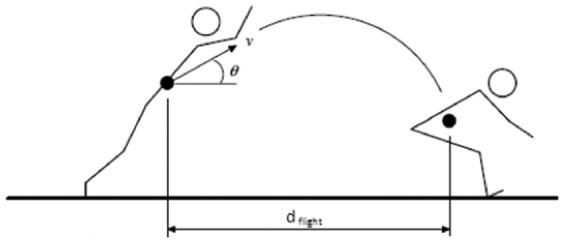
Depiction of standing horizontal jump, with key terms illustrated. *v*  =  take-off speed; *θ*  =  take-off angle, *d_flight_*  =  horizontal distance moved by the centre of mass of the body, *h_f_*  =  flight height (adapted from Wakai and Linthorne, 2005).

The take-off angle (the instantaneous projection of the COM at take-off, *θ*) was calculated using Eqn 2 (Hay, 1993), where flight height is the vertical distance the participant's COM travels from take-off to the peak height of the jump (Fig. 1).
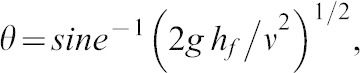
(2)where *θ*  =  take-off angle (degrees), *h_f_*  =  flight height (m), *v*  =  take-off speed (m s^−1^), and *g*  =  acceleration due to gravity (m s^−2^).

Descriptive statistics are presented as means ± 1 s.e.m. A general linear model with mL O_2_ m^−1^ as the dependent variable, jump condition as a fixed factor and participant identification as a random factor, including LSD post hoc tests, tested for differences between the four 1.8 m jump conditions. A separate general linear model with the same factors tested for differences between the three 1.2 m jump conditions. Paired t tests were used to test for differences in the kinematic variables between selected jump conditions. 95% confidence intervals (CI) of these outputs are reported alongside the p values where appropriate (Colegrave and Ruxton, 2003).

## RESULTS

**Fig. 2.**
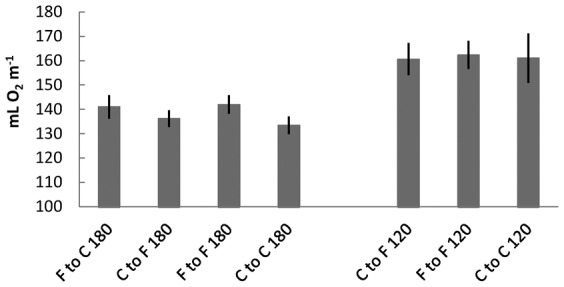
Mean oxygen consumption per metre (mL O_2_ m^−1^) of jump distance between springboards during horizontal jumping. Seven jumping conditions were generated by varying jumping distance (±one s.e.m.) (1.8 or 1.2 m) and the springboard type jumped from and jumped to (F  =  firm; C  =  compliant).

Data were confirmed for reasonable normality and homogeneity of variance. The general linear model for the mL O_2_ m^−1^ data for 1.8 m jumps indicated a strongly statistically significant effect of jump condition (F_3,56_ = 8.980, p<0.001; Fig. 2). For jumps of 1.8 m distance, jumping from a compliant springboard to another compliant springboard resulted in the lowest energy expenditure (133.5±3.6 mL O_2_ m^−1^). There was some evidence that this was lower than for jumping from a compliant springboard to a firm springboard (136.2±3.5 mL O_2_ m^−1^; p = 0.051; CI = −7.8 to 0.0). Further, jumping from a compliant springboard to a firm springboard resulted in a lower energy expenditure than when jumping from a firm springboard to another firm springboard (142.0±3.8 mL O_2_ m^−1^; p = 0.004; CI = −9.6 to −1.9) and there is some evidence that the former also resulted in a lower energy expenditure than when jumping from a firm springboard to a compliant one (141.1±4.8 mL O_2_ m^−1^; p = 0.063; CI = −7.9 to 0.2). Jumping from a compliant springboard to another compliant springboard was also less energetically costly than jumping in either the firm to firm condition (p<0.001; CI = −13.7 to −5.6) or the firm to compliant condition (p<0.001; CI = −11.8 to −3.6). There was no difference in energy expenditure for jumping in the firm to firm condition compared to the firm to compliant condition (p = 0.368; CI = −2.3 to 6.0).

The general linear model for the mL O_2_ m^−1^ data for 1.2 m jumps indicated there was no effect of jump condition (F_2,24_ = 0.261, p = 0.772; Fig. 2); energy expenditure for jumps of 1.2 m was similar across all conditions. Jumping from a compliant springboard to another compliant springboard (161.0±10.2 mL O_2_ m^−1^) was similar energetically to jumping from a compliant to a firm springboard (160.6±6.6 mL O_2_ m^−1^; p = 0.777; CI = −9.5 to 7.2) and also similar to jumping between firm springboards (162.3±5.7 mL O_2_ m^−1^; p = 0.490; CI = −5.5 to 11.2). The energy expenditure jumping from a compliant to a firm springboard was also similar to the energy expenditure for jumping between firm springboards (p = 0.644; CI = −9.1 to 5.7).

Considering together the range of pairwise post hoc comparisons for the 1.8 m jumps, there is considerable evidence that jumping from the compliant springboard is energetically less costly than jumping from the firm springboard. While there is also some evidence that landing on a compliant springboard is less energetically costly than landing on a firm one, it is weaker and the pattern is inconsistent. There is very little evidence that the energy costs of 1.2 m jumps are affected by jumping condition. Therefore, further analysis and discussion focusses on trying to uncover and interpret why jumps from compliant springboards are less energetically costly than from firm springboards at 1.8 m but are not less energetically costly at 1.2 m.

Analysis of the kinematic variables was undertaken to explain the differences found in energy expenditure between jumping conditions. Inferential statistical analysis of the kinematic variables therefore focussed firstly on testing for pertinent differences between 1.8 m jumps from a firm surface (i.e. amalgamating the conditions firm to firm and firm to compliant; firm surface take-off conditions) and from a compliant surface (compliant surface take-off conditions). Secondly, kinematic variables that were significantly different between firm surface take-offs and compliant surface take-offs at 1.8 m were then investigated for the conditions representing 1.2 m jumps, again comparing firm surface take-offs with compliant surface take-offs. Mean values for all kinematic measurements are provided in Table 1.

**Table 1.**
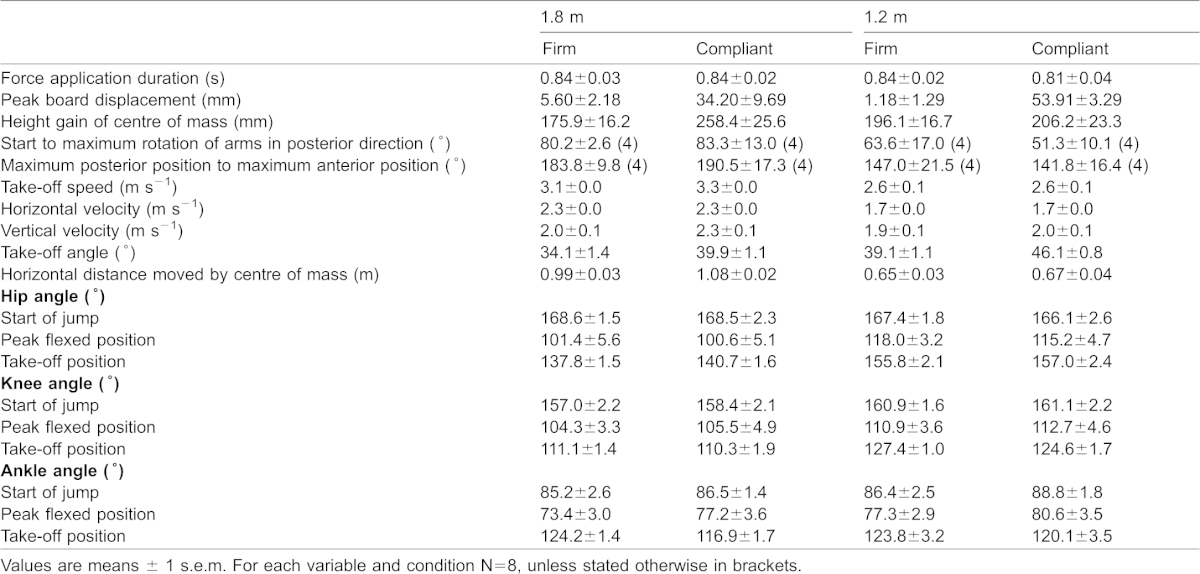
Kinematic data of participants jumping horizontal distance of 1.8 and 1.2 m from springboards with different material properties (‘firm’ and ‘compliant’)

The duration of force application between the two 1.8 m jump take-off conditions was similar (t_7_ = 0.312; p = 0.765; CI = −0.03 to 0.04). However, greater peak height of the COM was achieved when jumping a horizontal distance of 1.8 m from the compliant springboard than when jumping the same horizontal distance from the firm springboard (t_7_ = 5.919; p = 0.001; CI = 49.55 to 115.49). There was also evidence that peak height achieved by the COM was greater when jumping from the compliant springboard a distance of 1.2 m (t_7_ = 2.879; p = 0.028; CI = 7.93 to 80.88).

The sweep of the arms was similar between the two 1.8 m jump take-off conditions, both in terms of the angle from the start to the maximum posterior position (t_3_ = 0.266; p = 0.808; CI = −29.04 to 35.28) and the angle from the maximum posterior position to the maximum anterior position (t_3_ = 0.544; p = 0.624; CI = −27.18 to 40.64).

Joint angles at the start of the jump were similar between the two 1.8 m jump take-off conditions (hip: t_7_ = 0.027; p = 0.979; CI = −7.03 to 7.20; knee: t_7_ = 0.867; p = 0.415; CI = −2.48 to 5.35; ankle: t_7_ = 0.537; p = 0.608; CI = −4.33 to 6.87). Joint angles at peak flexed position were also similar (hip: t_7_ = 0.180; p = 0.862; CI = −10.02 to 11.67; knee: t_7_ = 0.397; p = 0.703; CI = −5.71 to 8.01; ankle: t_7_ = 1.623; p = 0.149; CI = 1.75 to 9.40). However, at the point of take-off, while the joint angle of the knee was similar (t_7_ = 0.845; p = 0.426; CI = −1.51 to 3.19), the joint angle of the hip was greater when jumping from a compliant springboard (t_7_ = 2.539; p = 0.039; CI = 0.20 to 5.60) while the joint angle of the ankle was smaller (t_7_ = 4.073; p = 0.005; CI = 3.04 to 11.47). Joint angle measurements were similar between the two 1.2 m jump take-off conditions at the start of the jump (hip: t_7_ = 0.716; p = 0.497; CI = −3.03 to 5.67; knee: t_7_ = 0.138; p = 0.894; CI = −3.68 to 4.14; ankle: t_7_ = 1.695; p = 0.134; CI = −0.95 to 5.74), at the peak flexed position (hip: t_7_ = 1.073; p = 0.319; CI = −3.42 to 9.11; knee: t_7_ = 0.895; p = 0.400; CI = −2.82 to 6.26; ankle: t_7_ = 1.206; p = 0.267; CI = −3.12 to 9.61) and also at the point of take-off (hip: t_7_ = 0.961; p = 0.369; CI = −1.84 to 4.36; ankle: t_7_ = 1.436; p = 0.194; CI = −2.40 to 9.83), except for some evidence that the knee at take-off is more flexed on the firm springboard (t_7_ = 2.140; p = 0.070; CI = −0.30 to 6.00).

Take-off speed from the firm springboard when jumping 1.8 m was significantly lower than from the compliant springboard (t_7_ = 3.614; p = 0.009; CI = 0.06 to 0.30), represented by a lower vertical velocity component (t_7_ = 2.917; p = 0.022; CI = 0.04 to 0.43) but not a different horizontal velocity component (t_7_ = 0.727; p = 0.491; CI = −0.07 to 0.14). This was associated with a slightly lesser horizontal distance covered by the COM (t_7_ = 3.134; p = 0.017; CI = 0.02 to 0.17). Take-off angle from the firm springboard when jumping 1.8 m was also lower (t_7_ = 5.119; p = 0.001; CI = 3.13 to 8.51). At 1.2 m, take-off angle was again lower from the firm springboard (t_7_ = 5.649; p = 0.001; CI = 4.03 to 9.82); however, take-off speed for 1.2 m jumps was similar between the two springboard types (t_7_ = 0.597; p = 0.569; CI = −0.10 to 0.17), as were both the horizontal velocity component (t_7_ = 0.109; p = 0.916; CI = −0.10 to 0.11) and the vertical component (t_7_ = 0.637; p = 0.544; CI = −0.12 to 0.22). There was no difference in the horizontal component covered by the COM (t_7_ = 0.951; p = 0.373; CI = −0.04 to 0.08).

## DISCUSSION

The mean amount of oxygen consumed to jump 1.8 m horizontally by the participants in the present study averaged around 249 mL, representing an energy expenditure of around 4.9 kJ (1.2 kcal) given certain assumptions (Walsberg and Hoffman, 2005). Horizontal jumps of 1.2 m consumed on average around 194 mL of oxygen (3.8 kJ, 0.9 kcal). In comparison, the cost for a person to walk 1 m on the flat is about 0.17 kJ (0.04 kcal) and to run 1 m is about 0.25 kJ (0.06 kcal) (Halsey and White, 2012), while walking 1 m on an incline of 12° expends around 0.62 kJ (0.15 kcal) (Halsey et al., 2008). Although measures of energy expenditure during pedestrian locomotion involve relatively consistent forward motion maintaining momentum, in contrast to the intermittent horizontal jumping in the experimental design of the present study, nonetheless, and as would be expected, these values indicate that for humans, horizontal jumping is a very energy expensive form of locomotion; per unit distance around 18-fold the cost to walk and around 12-fold the cost to run.

While at the shorter jump distance there was no apparent difference in energy expenditure between jumping conditions, there was good evidence that jumps of 1.8 m were energetically cheaper when take-off was from a compliant springboard (Fig. 2). Due to the difference in compressibility of the firm and compliant springboards, we hypothesised that the force application time required to jump from the compliant springboard would be greater than from the firm springboard. In turn, the slower force application would be more muscularly efficient and explain the decreased cost of jumping from a compliant surface (Aziz and Teh, 2005; Kang et al., 2004). However, there was no difference in the duration of force application between conditions. Instead, there may be a difference in muscle employment between take-off conditions for 1.8 m jumps. At the point of take-off during a jump (as the feet lose contact with the ground) the hips are more flexed and the ankles more extended when jumping from the firm springboard. This may indicate that 1.8 m jumps from the firm springboard in particular employ the gastrocnemius more and the thigh muscles less than equivalent length jumps from the compliant springboard. In turn, we conjecture that the gastrocnemius may produce the necessary force to jump less energetically efficiently than the thigh due to the former's shorter fascicle length requiring the necessary force to be produced more quickly. In turn this could help explain the greater energy cost to jump from the firm springboard. These differences in joint angles were not apparent between springboard types at 1.2 m; evidence that whatever the underlying reason, the joint angle differences observed at 1.8 m at least partially explain the differences in jumping cost at that distance.

However, perhaps the strongest evidence to explain the energetic difference between 1.8 m jumps comes from variation in take-off angle. The energy produced by the impulse required to jump, calculated from first principles, can be found using Eqn 3 (Bennet-Clark, 1975).
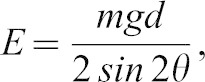
(3)where *E*  =  energy produced by the impulse (J), *g*  =  acceleration due to gravity (m s^−2^), *m*  =  mass (kg), *d*  =  jump distance (m), and *θ*  =  take-off angle (degrees).

The peak value for a sine wave is 1, which occurs for an angle of 90°. Based on Eqn 3, where the sine is taken for the take-off angle multiplied by 2, the theoretical optimum take-off angle for minimising the energy produced by the impulse of a projectile will be 45°. The jump distance can be found using Eqn 2; however, as jump angles increase participants must spend a greater fraction of their muscular force overcoming body weight and so the take-off speed will decrease. Therefore the optimum take-off angle for achieving optimum jump distance in a standing long jump from a firm surface has been calculated as (19–27°) and measured experimentally at 31–39° (Eqn 4) (Wakai and Linthorne, 2005).
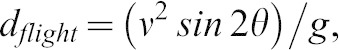
(4)where *d_flight_*  =  distance travelled while air borne (m), *v*  =  take-off speed (m/s), *θ*  =  take-off angle (degrees), and *g*  =  acceleration due to gravity (m/s^2^).

For the 1.8 m jumps the take-off angle was greater when jumping from compliant springboards (40±1.1°) than from firm surfaces (34±1.4°). Thus the compliant surface allows the required (greater) take-off speed to be achieved despite the increased angle, presumably due to the impulse provided by the springboard as it returns stored energy during the final stages of the take-off. Present data cannot ascertain if the increased take-off angle is at the volition of the participant and/or the result of the near vertical movement of the compliant springboard as it returns after compression. The increase in take-off angle, and necessarily therefore take-off speed, led to a significantly higher peak COM height during the flight phase when jumping from the compliant surface. The associated higher gravitational potential energy intuitively suggests that the activity would be more costly, in contrast to the present findings. Thus the reduction in energetic cost when jumping from a compliant surface appears to be at least in part a result of the increase in the take-off angle, which is thus closer to the theoretical optimum for reducing the energy cost produced by the impulse, i.e. the cost to jump based on first principles. Indeed, this closer-to-optimal take-off angle from the compliant springboard, perhaps in concert with different leg muscle utilisation, more than compensates for the increased cost associated with raising the COM a greater vertical height.

We hypothesised that the energetic savings achieved when jumping from a compliant springboard in comparison to a firm springboard would be greater when jumping greater distances. Our findings support this by demonstrating a reduction in the cost to jump when using a compliant springboard for take-off in comparison to a firm springboard for the 1.8 m jumps but not for the 1.2 m jumps. However, the mechanism for this difference appears not to be due to the difference in the capacity to utilise the board compliance between the two different jump distances. While the costs for 1.2 m jumps did not differ between conditions, nonetheless, similarly to the 1.8 m jumps a significant difference was observed for the angle of take-off when comparing jumping from a firm surface (39±1.1°) to jumping from a compliant surface (46±0.8°), and indeed peak vertical height gained by the COM, although in this case there was no difference in take-off speed. This suggests the participants were still able to utilise the board compliance despite the lower energy required to achieve the jump at 1.2 m. Interestingly, for both springboard types the take-off angle was improved towards the theoretical optimum of 45° by 5–6° when jumping 1.2 compared to 1.8 m. This was presumably made possible due to the lower take-off speed required to achieve the shorter horizontal distance. An explanation as to why a significant difference in the cost to jump is observed between the different springboard types over the 1.8 m jumps only, despite the similar difference in take-off angle between the firm and compliant springboards at both jumping distances, may be found from Eqn 4. The shape of a sine wave is such that its gradient varies from its maximum at 0° (gradient  =  1) to its minimum at 90° (gradient  =  0). Therefore the energy cost produced by the impulse is far less sensitive to changes in the take-off angle the closer the angle is to the theoretical optimum of 45°. For the 1.2 m jumps, despite the take-off angle being closer to the theoretical optimum when jumping from a compliant surface than when jumping from a firm surface, this increase in angle occurs over a less sensitive area of the sine curve (close to *2θ* = 90°), than the change in angle for the 1.8 m jumps. The theoretical reduction in energy cost produced by the impulse associated with the change in observed take-off angle when comparing jumping from the firm surface and jumping from the compliant surface is 2% for jump distances of 1.2 m. The reduction in the energy cost produced by the impulse associated with the observed change in take-off angle is 6% for the same comparison for 1.8 m jumps. This may explain why statistically significant increases in take-off angle with a similar magnitude results in a reduction in energetic cost when jumping 1.8 m but not when jumping 1.2 m.

Finally, we hypothesised that landing on a compliant support would be more energetically efficient than landing on a firm support; however, the findings of this study did not lend clear evidence for this prediction. The reason for this may be that our compliant support had a very low damping ratio (0.007), therefore despite the board initially absorbing a great deal of the participant's kinetic energy, much of this energy was returned by the board. This energy then had to be absorbed by the participant's musculoskeletal system. Possibly a highly damped landing surface would more clearly support our prediction.

To summarise, this study has found compelling evidence that horizontal jumping from a compliant substrate is energetically less costly than horizontal jumping from a firm substrate over longer distances, although the effect size is fairly small within the range of compliances tested; about a 6% difference in energy expenditure. Evidence for an effect on jumping energy costs due the properties of the landing substrate was less clear, and there was no evidence for an effect of either take-off or landing substrate type at shorter distances. The main explanation for the effect of take-off substrate type at longer jumping distances is the angle of take-off that can be achieved, with angles closer to the theoretical optimum of 45° exhibited when taking off from a compliant substrate. This more acute angle can presumably be utilised because sufficient speed can nonetheless be obtained due to the impulse provided by the springboard as it returns stored energy during the final stages of the take-off. These findings are also likely relevant to at least other jumping primates, which will typically experience a great variety of take-off and landing substrates (Alexander, 1991; Demes et al., 1995; Channon et al., 2011). There are of course many substrates with other material properties that could be jumped between to further understand the energy expenditure of horizontal jumping, both in humans and other species (cf. Bonser et al., 1999), and how the take-off and landing environments affect these costs and the associated changes in kinematics and biomechanics that underlie them.

## Supplementary Material

Supplementary Material
