# Supplementary material for: Energy expended during horizontal jumping: investigating the effects of surface compliance
Source: Biol Open. 2014 Aug 22;3(9):815–20. doi: 10.1242/bio.20148672 (PMC4163658; doi:10.1242/bio.20148672)
Supplement: Supplementary Material [file supp_3_9_815__index.html]

Energy expended during horizontal jumping: investigating the effects of surface compliance — Energy expended during horizontal jumping: investigating the effects of surface compliance — Supplementary Material 

# Energy expended during horizontal jumping: investigating the effects of surface compliance

## bio.20148672 Supplementary Material

**Files in this Data Supplement:**

- Supplementary Material - Samuel R. L. Coward and Lewis G. Halsey doi: 10.1242/bio.20148672
