# Supplementary material for: Energy expended during horizontal jumping: investigating the effects of surface compliance
Source: Biol Open. 2014 Aug 22;3(9):815–20. doi: 10.1242/bio.20148672 (PMC4163658; doi:10.1242/bio.20148672)
Supplement: Supplementary Material [file supp_bio.20148672_bio.20148672-s1.pdf]

Biology Open (2014) 000, 1–6 doi:10.1242/bio.20148672

Supplementary Material

Samuel R. L. Coward and Lewis G. Halsey doi: 10.1242/bio.20148672

Fig. S1. Damping ratio and stiffness calculations

Calculation of damping ratio for springboards

A drop test was performed (Nigg and Yeadon, 1987) in which a shot was dropped onto the two different surfaces (compliant springboard, firm springboard) from a height of approximately 0.5 m. The shot had the following properties: steel, mass = 26.7 kg, radius = 83 mm. The test was recorded using a high speed camera (Promon Pro, AOS, Switzerland) at 200 Hz and was repeated five times on two replicates of each surface type. The damping ratio was determined using Eqn S1 and Eqn S2 (Nagurka and Huang, 2006).

$$e=\left|\frac{\dot{x}(\Delta T)}{\dot{x}(0)}\right| = \left|\frac{v_i}{v_0}\right|, \text{(S1)}$$

where e = coefficient of restitution, v_i = post impact velocity (m s^{-1}), and v_0 = pre impact velocity (m s^{-1}).

$$\zeta=-\frac{ln\, e}{\sqrt{\pi^2+(ln\, e)^2}}, \text{(S2)}$$

where ζ = damping ratio.

Calculation of stiffness of springboards

The height of the top surface of the springboard was measured with the board unloaded. The board was then loaded with a known mass (92.4 kg) and the height of the top surface under load was then measured. The board stiffness was then calculated using Eqn S3 (Nigg and Yeadon, 1987).

$$F=k(l-l_o), \text{(S3)}$$

where F = applied force (N), k = stiffness (N m^{-1}), l = original height of surface (m), and l_o = height of surface under load (m).

Biology Open
